# *Leptospira noguchii* and Human and Animal Leptospirosis, Southern Brazil

**DOI:** 10.3201/eid1504.071669

**Published:** 2009-04

**Authors:** Éverton F. Silva, Gustavo M. Cerqueira, Núbia Seyffert, Fabiana K. Seixas, Daiane D. Hartwig, Daniel A. Athanazio, Luciano S. Pinto, Adriano Queiroz, Albert I. Ko, Claudiomar S. Brod, Odir A. Dellagostin

**Affiliations:** Universidade Federal de Pelotas, Pelotas, Brazil (É.F. Silva, G.M. Cerqueira, N. Seyffert, F.K. Seixas, D.D. Hartwig, L.S. Pinto, C.S. Brod, O.A. Dellagostin); Universidade Federal da Bahia, Salvador, Brazil (D.A. Athanazio); Fundação Oswaldo Cruz, Salvador (D.A. Athanazio, A. Queiroz, A.I. Ko); Weill Medical College of Cornell University, New York, New York, USA (A.I. Ko)

**Keywords:** Zoonoses, Leptospira noguchii, leptospirosis, isolation, taxonomy, letter

**To the Editor:** Pathogenic leptospires, the causative agents of leptospirosis, exhibit wide phenotypic and genotypic variations. They are currently classified into 17 species and >200 serovars (*1*,*2*). Most reported cases of leptospirosis in Brazil are of urban origin and caused by *Leptospira interrogans* (*3*). Brazil underwent a dramatic demographic transformation due to uncontrolled growth of urban centers during the last 60 years. Urban slums are sites of poor sanitation that favors rat-borne transmission of leptospirosis among humans. Thus, this may explain the major involvement of serovar Copenhageni (*L. interrogans*). The predominance of *L. interrogans* is likely due to the underestimation of rural cases of leptospirosis.

Pelotas is a coastal city in Rio Grande do Sul State, in southern Brazil, with ≈400,000 inhabitants. This state has a typical temperate climate. However, the incidence of human leptospirosis is high (12.5/100,000 inhabitants in 2001) compared with the mean incidence in areas of Brazil where tropical and subtropical climates predominate (3.5/100,000 in the same year). Most cases in Rio Grande do Sul State (69%) occur in rural areas where the spatial distribution suggests an association with areas of rice field activities. Pelotas had an annual incidence of >50 cases per 100,000 inhabitants in 2001, which placed it among the cities with the highest incidence of leptospirosis in southern Brazil (*4*).

Before 2007, pathogenic serovars and strains in Brazilian collections included the following species: *L. santarosai*, *L. interrogans*, *L. kirshneri,* and *L. borgpetersenii*. However, our research group has recently reported the isolation of *L. noguchii* in Brazil from sheep (*5*). This species had been previously isolated from animals such as armadillo, toad, spiny rat, opossum, nutria, the least weasel (*Mustela nivalis)*, cattle, and the oriental fire-bellied toad (*Bombina orientalis)* in Argentina, Peru, Panama, Barbados, Nicaragua, and the United States (*1*,*6*). Human leptospirosis associated with *L. noguchii* has been reported only in the United States, Peru, and Panama, with the isolation of strains Autumnalis Fort Bragg, Tarassovi Bac 1376, and Undesignated 2050, respectively (*1*,*6*). The Fort Bragg strain was isolated during an outbreak among troops at Fort Bragg, North Carolina. It was identified as the causative agent of an illness characterized by fever, headache, myalgia, and a pretibial rash—Fort Bragg fever (*7*). We were not able to obtain data regarding the other 2 human isolates.

We report the isolation of 3 additional *L. noguchii* strains from Brazil, including 2 from cases of human leptospirosis. The first isolate (Bonito strain) was obtained from the blood culture of a 34-year-old man who exhibited fever, headache, myalgia, hemorrhages, jaundice, abdominal pain, diarrhea, and vomiting. The patient reported contact with rats, farm animals, and dogs before the onset of illness. Laboratory tests at admission to the Hospital Santa Casa de Misericórdia, Pelotas, showed an elevated level of serum bilirubin (total 21 mg/dL, direct 16 mg/dL) and a slight increase in liver enzyme levels (alanine aminotransferase 2×, aspartate aminotransferase 1.5× above reference levels). An acute-phase serum sample showed a titer of 25 titer against serovars Autumnalis and Bratislava by microscopic agglutination test (MAT).

The second isolate (Cascata strain) was obtained from the blood culture of a 16-year-old boy who exhibited headache, fever, flulike symptoms, and myalgia. He reported previous contact with rats and dogs. The patient was not hospitalized, and an acute-phase serum sample showed a titer of 25 against saprophytic serovar Andamana by MAT. Both patients were from the rural area of Pelotas. Unfortunately, convalescent-phase serum samples were not obtained from these patients.

A third isolate (Hook strain) was obtained from a male stray dog with anorexia, lethargy, weight loss, disorientation, diarrhea, and vomiting. The animal died as a consequence of the disease. The isolate was obtained from a kidney tissue culture. No temporal or spatial relationship was found between the 3 cases.

Serogrouping was performed by using a panel of rabbit antisera. Bonito, Cascata, and Hook strains were classified as Autumnalis, Bataviae, and Australis, respectively. Serogroups were confirmed by the strong and specific reaction of hyperimmune sera against these isolates, with the reference strains of the respective serogroups. Species identification was accomplished by sequencing nearly the full length of the 16S rRNA gene, as previously described (*5*). The sequences of the Hook, Cascata, and Bonito strains were deposited in GenBank under accession nos. EU349494–EU349496.

In addition, the *rpoB* gene sequence was determined and used for further confirmation of the species. The *rpoB* sequence for the strains Hook, Cascata, Bonito, and the *L. noguchii* reference strains were deposited in GenBank under accession nos. EU349497–EU349505. BLAST (www.ncbi.nlm.nih.gov/blast/Blast.cgi) alignment confirmed the new isolates as *L. noguchii*. The 16S rRNA gene sequence was also used for taxonomic analysis of *L. noguchii* (Figure). The topology-based dendrogram demonstrates sequence relatedness among strains isolated in Pelotas and the *L. noguchii* Autumnalis, Australis, and Bataviae strains deposited in GenBank (Figure). No molecular or serologic characterization at the serovar level was performed.

**Figure:**
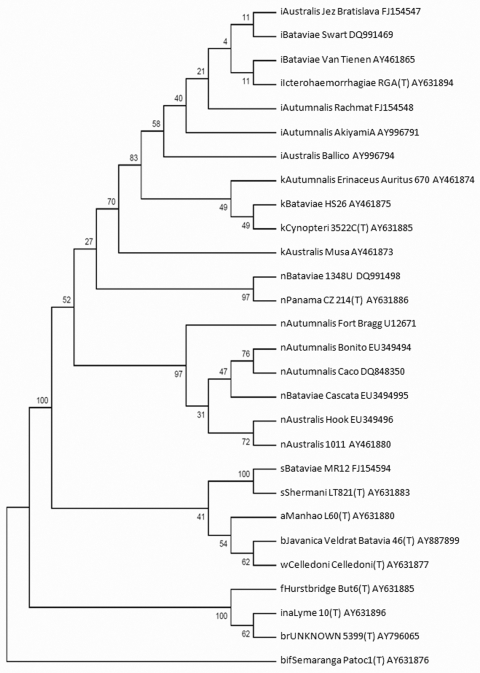
Dendogram constructed by using the neighbor-joining algorithm, based on a 1,180-bp sequence of the 16S rRNA gene demonstrating the position of the Brazilian strains (Bonito, Cascata, and Hook) within the *Leptospira noguchii* species. This dendogram summarizes, by bootstrap-based topology, the evolutionary relationship among *L. noguchii* strains. The bootstrap consensus values are indicated over each root. The initial lowercase letters indicate the respective species to which each strain belongs: i, *L. interrogans*; k, *L. kirschneri*; n, *L. noguchii*; b, *L. borgpetersenii*; w, *L. weilii*; s, *L. santarosai*; a, *L. alexanderi*; f, *L. fainei*; in, *L. inadai*; br, *L. broomi*; bif, *L. biflexa*. (T) indicates the type-strain for each species. The GenBank accession number follows the strain identification.

We report herein the occurrence of *L. noguchii* species in southern Brazil. The 3 isolates obtained belong to distinct serogroups. Information presented here places *L. noguchii* among the prevalent *Leptospira* species that are able to cause human and animal leptospirosis in southern Brazil.
